# Fellow-Workers and the Roll of Honour

**Published:** 1914-10-24

**Authors:** 


### ROUND THE WORLD.

[The Editor Invites Early Information and Contributions Marked "Fellow-Workers."]

### Fellow-Workers and the Roll of Honour.

The medical profession in London has been greatly complimented by several recent appointments of eminent members to act as consulting physicians and surgeons on the staff of the Expeditionary Force. It has scarcely been surprising that Sir Almroth Wright should have been included in this list, as his experience and profound knowledge not only of preventive vaccination against sepsis but of anti-typhoid inoculation, must render his services invaluable to any army in the field. This is, of course, by no means his first association with things military, as he was Professor of Pathology in the Army Medical School at Netley from 1892 to 1902, and his researches have vastly aided the Army doctors at any time during the last twenty years. Colonel Sir Almroth Wright, as he will now be, has carried out a remarkable amount of first-class work since he became Director of the Therapeutic Inoculation Department at St. Mary's Hospital some twelve years ago.

Sir Wilmot Parker Herringham, M.D., F.R.C.P., who likewise becomes a colonel on the Army medical staff in France, is well-known in London not only as a consulting physician attached to St. Bartholomew's Hospital, but as Vice-Chancellor of London University, and also as the successful Secretary of the recent International Congress of Medicine. A prolific writer on medical subjects, Sir Wilmot Herringham has found much pleasure and recreation in travelling and as an angler.

Sir John Rose Bradford, who will be the colleague of the two last-named physicians with similar rank, is a prominent member of the medical staff at University College Hospital, and has also been closely associated with London University, where he was at one time a member of the Senate. Ever since his student days and the time when he won the gold medal at the M.D. London examination some twenty-five years ago, Sir John Rose Bradford has had a very distinguished career. His present appointments include those of physician to the Seamen's Hospital at Greenwich, member of the Advisory Board of the Army Medical Service, and senior medical adviser to the Colonial Office.

Sir Alfred Keogh, K.C.B., is to be congratulated on his resumption of the duties of Director-General of the Army Medical Department at home, an office which he is again taking up now that Sir Arthur Sloggett is going abroad as Director-General of the Expeditionary Force. Sir Alfred Keogh, who has lately been doing such excellent work as the chief commissioner of the British Red Cross in France, was, it will be remembered, Director-General from 1904 to 1909, when he did much to promote the efficiency of the important service under his control, as well as to secure the interests of its officers. A student of "Guy's" and Queen's College, Galway, Sir Alfred Keogh entered the Army soon after qualification, and in the course of his career has held many important administrative posts. At the time of the South African war he had charge of a general hospital, which he administered with notable distinction. He was made a C.B. in 1900, and promoted to K.C.B. six years later.

Sir Anthony Alfred Bowlby, F.R.C.S., who goes out as colonel on the staff of the Expeditionary Force, with special duties as consulting surgeon, is one of our most distinguished operators and a member of the staff of St. Bartholomew's Hospital, as well as surgeon-in-ordinary to his Majesty the King and consulting surgeon to Queen Alexandra's Military Hospital. Colonel Sir Anthony Bowlby served in the South African war in 1900, where he was in charge of the Portland Hospital, and in addition to being mentioned in dispatches, was made a C.M.G. He was knighted three years ago.

Mr. George Henry Makins, C.B., F.R.C.S., who will be Colonel Sir Anthony Bowlby's colleague at the front, is surgeon to St. Thomas's Hospital, consulting surgeon to the Evelina Hospital, and Vice-President of the Royal College of Surgeons. He has for many years been closely associated with the Army Medical Department, and was consulting surgeon to the South African Field Force (1899-1900), when he had many interesting experiences subsequently embodied in a book he wrote on returning home. Mr. Makins, who was decorated for his services in South Africa, has also been President of the Board of Examiners of the Naval Medical Service, and an examiner both to the R.A.M.C. and I.M.S.

Lieutenant-Colonel Eustace M. Callender, who is in command of the 2nd London (City of London) General Hospital, is in private life a busy general practitioner in the West End of London, where he has been established for many years. An old St. Mary's man, he qualified M.R.C.S. in 1886, subsequently becoming an M.D. Brux. with highest distinctions. Formerly house physician to St. Mary's Hospital, his present appointments include those of physician to the Medical Aid Society, honorary medical officer to St. Agatha's Home, St. John's Wood, and honorary medical officer to the Working Ladies' Guild.

Major Sir Edward Scott Worthington, who has also left for the Front, has been for the last three years medical officer to the Governor-General of Canada, and had the responsible task of attending the Duchess of Connaught during her recent illness, when his duties, of course, included the care of her Royal Highness's health during the voyage to this country and afterwards on the return journey to Canada. Originally a student of Trinity College, Toronto, Sir Edward Worthington came to London and became an L.R.C.P., M.R.C.S. in 1897, entering the R.A.M.C. three years later. He served in the South African war with distinction, and was medical officer on the staff of the Duke of Connaught during the South African visit for the opening of the Union Parliament.

Mr. Charles Mansell Moullin, M.D., F.R.C.S., who has now been promoted to the rank of lieutenant-colonel in the 2nd (City of London) General Hospital of the Territorial establishment, is a well-known West-End consultant, and was for many years on the active staff of the London Hospital. In his early years he was a student of Pembroke College, Oxford, and St. Bartholomew's. At one time he examined in surgery for the universities of Oxford and Cambridge.

Colonel Michael William Russell, R.A.M.C., who has just been appointed an inspector of administration in connection with the medical and sanitary services, with special reference to the German prisoners' encampment, is an old Middlesex Hospital man, and qualified M.R.C.S., L.S.A. in 1881-82. In earlier years he was resident medical officer to the Royal United Hospital at Bath.

